# Eccrine Hidrocystoma of the Central Chest and Dermoscopic Findings

**DOI:** 10.7759/cureus.8012

**Published:** 2020-05-07

**Authors:** Anna M Zanot, Jaime Tschen, Sirunya Silapunt

**Affiliations:** 1 Dermatology, University of Texas McGovern Medical School, Houston, USA; 2 Dermatology, St. Joseph Dermatopathology, Houston, USA

**Keywords:** hidrocystoma, eccrine hidrocystoma, dermoscopy, dermoscopic findings, chest

## Abstract

Eccrine hidrocystomas are benign, cystic tumors that are most commonly found on the central face in middle-aged females. Their dermoscopic findings are rarely described in the literature, with only seven cases currently reported to date. We present the case of an elderly man with an unusual location of an eccrine hidrocystoma of the central chest and its associated dermoscopic findings. Characterizing the dermoscopic features of eccrine hidrocystomas may allow for better differentiation of these lesions from cutaneous malignancies and may minimize unnecessary biopsies, treatment, and scarring.

## Introduction

Hidrocystomas are benign, cystic tumors that arise from apocrine or eccrine sweat glands. Apocrine hidrocystomas present as translucent nodules that are commonly found on the eyelids and cheeks in middle-aged males and females [1,2]. Eccrine hidrocystomas present as single or multiple flesh-colored to blue, translucent papules that are worsened by perspiration, heat, and humidity and often regress in cold climates [3-5]. They are most commonly found on the central face and are most prevalent in middle-aged females [3,4]. Currently, few literatures describe the dermoscopic findings of eccrine hidrocystomas. Here we present the case of a 70-year-old man with an eccrine hidrocystoma on the central chest and its associated dermoscopic findings.

## Case presentation

**Figure 1:**
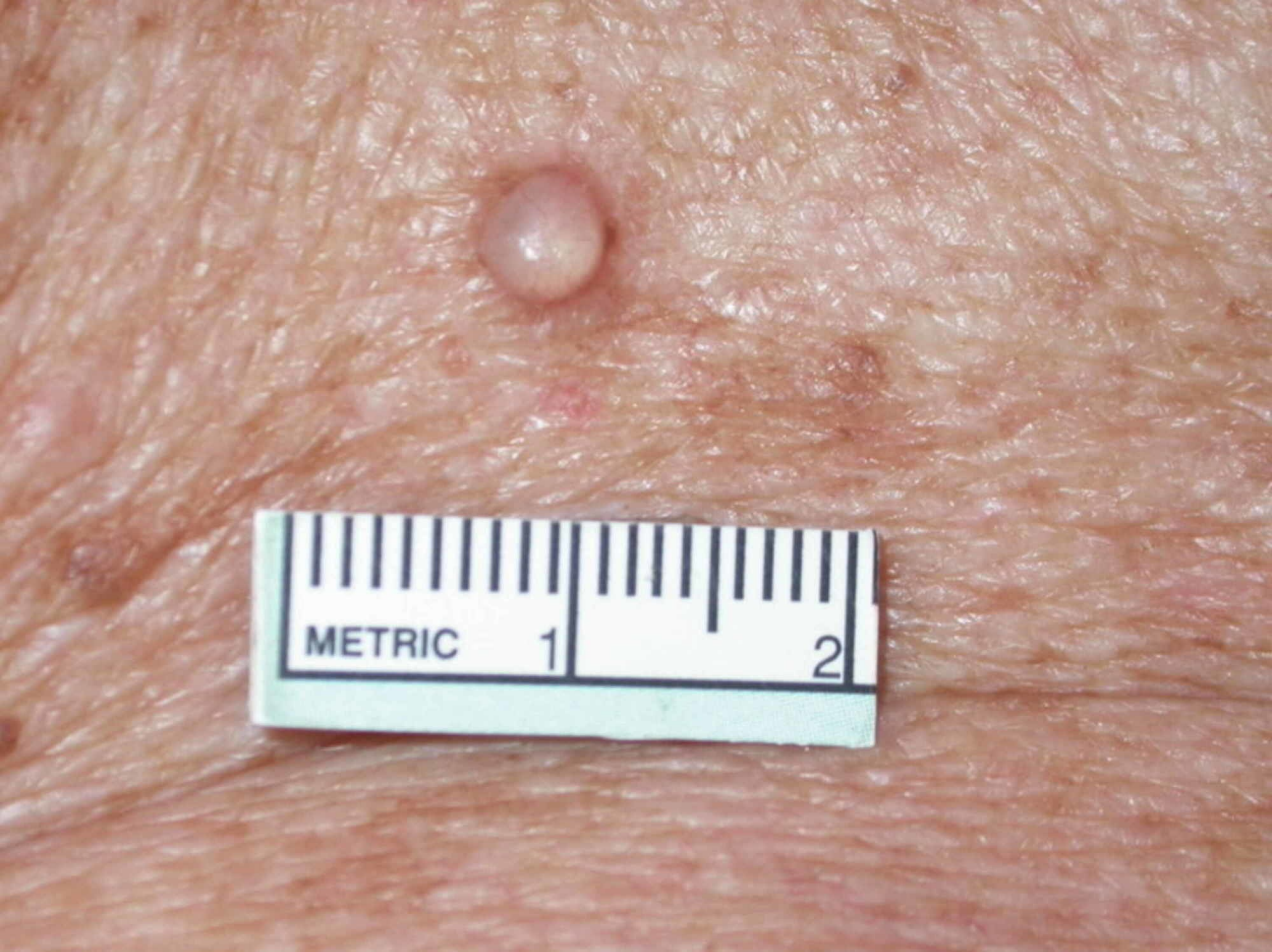
A well-defined, dome-shaped, cystic nodule on the central chest.

A 70-year-old man presented with a several month history of an asymptomatic growing lesion on his central chest. The patient denied personal and family history of skin cancer. Physical exam revealed a solitary, smooth surfaced, well-defined, dome-shaped, cystic nodule measuring 5 mm on the central chest (Figure 1).

**Figure 2:**
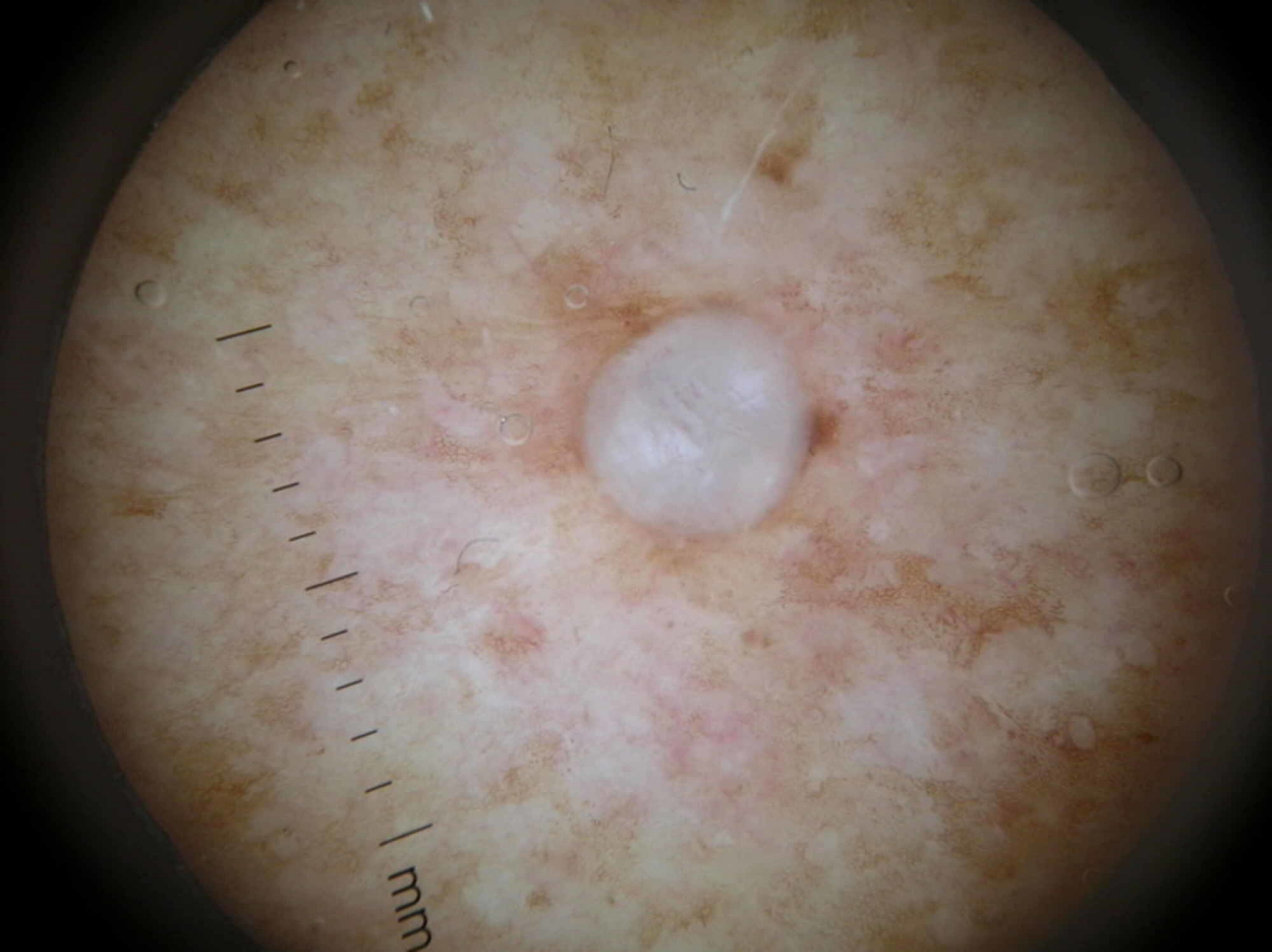
Dermoscopy revealed a well-demarcated, milky, ovoid lesion with a lentiginous background.

The differential diagnosis included hidrocystoma, cystic basal cell carcinoma, papular mucinosis, milia, and follicular tumors. On dermoscopy, a well-demarcated, cystic, milky lesion was visualized (Figure 2). 

**Figure 3:**
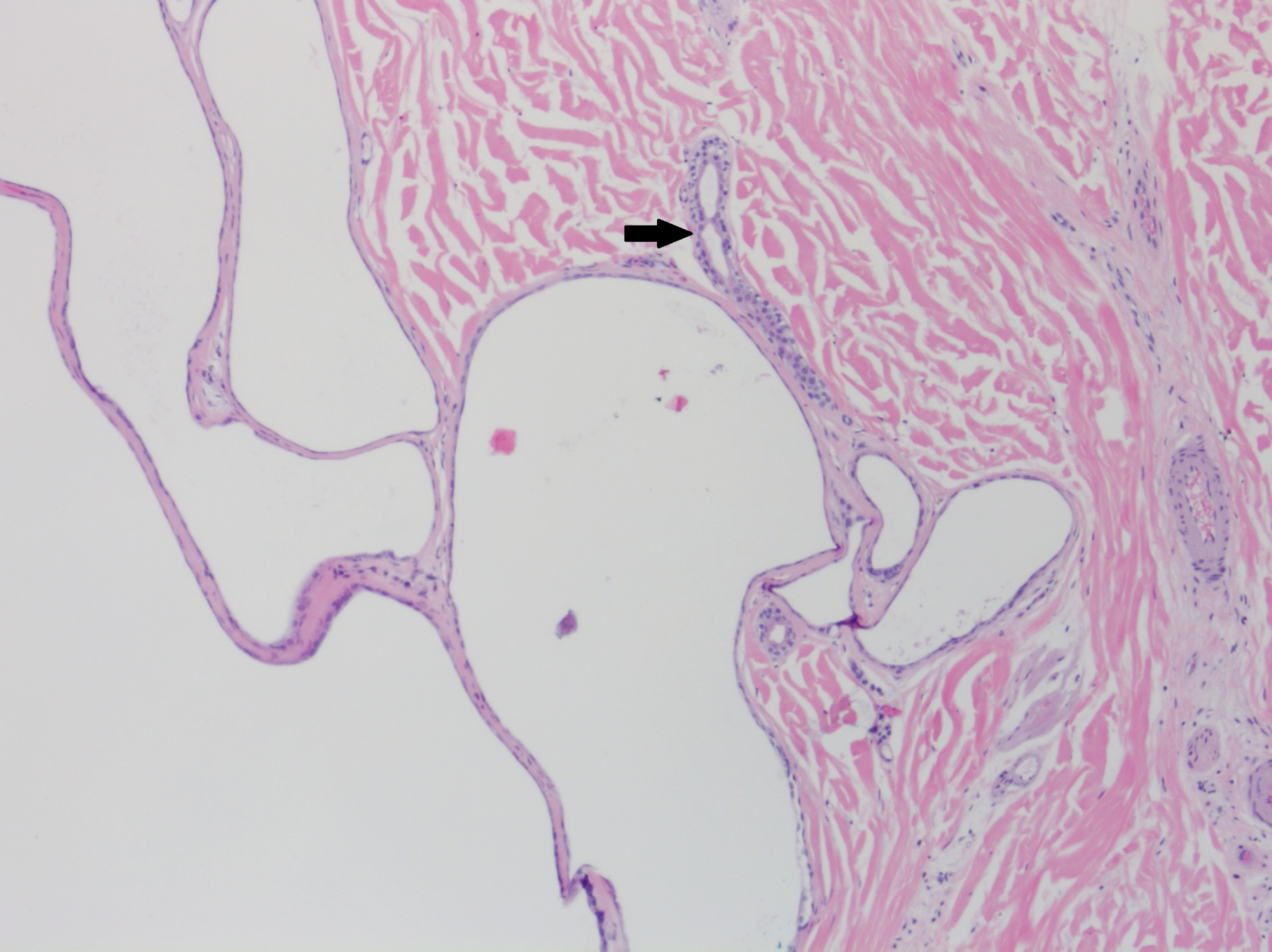
Histopathology revealed a dermal-located cyst lined by two layers of cuboidal epithelium. The cyst is multiloculated and is arising in continuity with an eccrine duct (arrow). Hematoxylin-eosin stain; original magnification: 100x.

A punch biopsy was performed to excise the whole lesion, and histopathology revealed a dermal-located cyst lined by two layers of cuboidal epithelium (Figure 3). Immunohistochemical stains were negative for gross cystic disease fluid protein-15 and mammaglobin. These findings were consistent with the diagnosis of eccrine hidrocystoma. There was no recurrence of the lesion at one-year follow-up. 

## Discussion

Our case expands on the clinical features of eccrine hidrocystomas and attempts to characterize their dermoscopic findings to assist in their correct diagnosis. Apart from one case on the breast, there are no other reports of eccrine hidrocystomas on the chest to the best of our knowledge [3]. Thus, we present an unusual location for these lesions. Eccrine hidrocystomas have primarily been described on the central face, particularly in the malar and periorbital regions [3,4]. There are few cases involving the scalp, vulva, and shoulder [6-8]. Clinicians should be aware of the variable locations of these lesions in formulating their differential diagnoses.

Eccrine hidrocystomas are usually slow growing and asymptomatic [4,9]. On gross examination, they are typically 1-6 mm in size and can be solitary or multiple, tense, thin-walled, dome-shaped, bluish, and cystic papules [3,4]. Their dermoscopic features are rarely described in the literature, with only seven cases currently reported to date [5,10-12]. Correia et al. reported the dermoscopic findings of an eccrine hidrocystoma to be cystic, avascular, and well demarcated [5]. Likewise, our dermoscopic findings revealed a well-demarcated, homogenous, milky, cystic papule. While a bluish hue was not observed in our case, most cases in the literature have reported this finding [3,4,10-12]. Lastly, pale halos surrounding bluish papules have been found under dermoscopy in four reported cases; however, this was not seen in our case [10,11]. 

Characterizing the dermoscopic features of eccrine hidrocystomas would allow for better differentiation of these lesions from cutaneous malignancies. Importantly, both eccrine hidrocystomas and cystic basal cell carcinoma are predominantly found on the head and neck and may have similar presentations. In contrast to our findings, the dermoscopic features of basal cell carcinoma consist of arborizing telangiectasias, ulceration, and blue/gray globules and nests [13]. Using dermoscopy to help distinguish eccrine hidrocystomas from basal cell carcinoma may minimize unnecessary biopsies, treatment, and scarring.

Currently, the diagnosis of eccrine hidrocystomas is confirmed via histologic examination [11]. Unlike eccrine hidrocystomas, apocrine hidrocystomas are characterized by secretory decapitation, periodic acid-Schiff (PAS)-positive granules, papillary projections, and columnar cells with eosinophilic cytoplasm [9,14]. Apocrine immunohistochemical stains, such as gross cystic disease fluid protein-15 and mammaglobin, may be used to assist in diagnosis, which were negative in our case. In addition, eccrine hidrocystomas have been found to stain S-100 positive and PAS negative [14]. Eccrine and apocrine hidrocystomas may both appear homogeneous on dermoscopy. Arborizing vessels, linear-irregular vessels, white streaks, and irregular brown pigment globules are commonly visualized in apocrine hidrocystomas and were not observed in our case [1,2,15].

While clinically asymptomatic and benign, eccrine hidrocystomas may be treated for cosmetic concerns or for visual impairment [16]. Intradermal botulinum toxin injections have led to successful resolution of these lesions of the face [5]. Other available treatments with varying efficacy include electrodesiccation, surgical excision, incision and drainage, pulsed dye laser treatment, and topical atropine and scopolamine [9,17-19]. Defining the dermoscopic features of eccrine hidrocystomas may assist in monitoring for recurrence and resolution following treatment.

## Conclusions

We report an unusual location of an eccrine hidrocystoma of the central chest and its rarely described dermoscopic findings. Clinicians should consider eccrine hidrocystomas in their differential diagnosis of homogenous, cystic papules of the central chest. In addition, dermoscopy may be utilized to help differentiate these lesions from cutaneous malignancies.
